# Effects of compost, biochar and ash mixed in till soil cover of mine tailings on plant growth and bioaccumulation of elements: A growing test in a greenhouse

**DOI:** 10.1016/j.heliyon.2022.e08838

**Published:** 2022-01-27

**Authors:** Juha Heiskanen, Hanna Ruhanen, Marleena Hagner

**Affiliations:** a Natural Resources Institute Finland (Luke), Soil Ecosystems, Juntintie 154, FI-77600 Suonenjoki, Finland; b Natural Resources Institute Finland (Luke), Experiment and Data Services, Juntintie 154, FI-77600 Suonenjoki, Finland; c Natural Resources Institute Finland (Luke), Plant Health, Tietotie 2C, FI-31600, Jokioinen, Finland; d Ecosystems and Environment Research Programme, Faculty of Biological and Environmental Sciences, University of Helsinki, 15140 Lahti, Finland

**Keywords:** Land reclamation, Mine closure, Recyclates, Reforestation, Vegetation restoration

## Abstract

Mine closures necessitate vegetation restoration to cover tailings fields and reduce environmental risks. Sole use of forest soil as growth medium provides only low fertility and slow plant growth especially in the harsh boreal climate conditions. This preliminary study examines the feasibility of recyclable waste materials added to forest till soil for improving vegetation success on reclaimed mine tailings. One compost type, three biochar types (Bc1–3) and two ash types (Ash1-2) were studied for physical and chemical properties as well as their effects on the growth and element accumulation of timothy (*Phleum pratense* L.), white clover (*Trifolium repens* L.) and Scots pine (*Pinus sylvestris* L.) during one growing period in a greenhouse. Oxidized surface tailings soil and Ash2 were the finest media components while compost and Ash1 were the coarsest. Tailings soil also had the highest salt contents and electrical conductivity, while in till soil they were at the lowest levels. Timothy and white clover germinated well in moist pure tailings soil but grew poorest in it. White clover grew poorly also in pure till soil. Best biomass growth was in the mixture of till, compost and Bc2 (from sewage sludge and woodchips). Planted pine seedlings grew relatively well in all media during the first growing season but Ash1 (from wood and peat) tended to promote height growth and pure till soil root biomass. In media containing Ash1, pine tissues accumulated B, Ca, Mg, K, Na and S. Elevated As content in tailings soil accumulated in plant shoot tissues slightly; only in the old needles of pine were As levels elevated. The results suggest that till and tailings media with compost added as a nitrogen source can promote adequate plant growth during initial growing seasons. Suitable types of biochar and ash amendments can further expedite plant establishment.

## Highlights

• White clover and timothy germinants showed poor growth in pure tailings soil.
• Adding 5 % compost to tailings soil promoted growth of these species.
• Biochar and ash addition into tailings and till expedited growth further.
• Planted Scots pine seedlings grew relatively well in all the growth media studied.
• Element bioaccumulation did not differ clearly among media or plant species.

## 1 Introduction

Mine closures after ore-extracting activities need landscape reclamation to reduce the environmental disadvantages associated with tailings fields. To minimize the release of harmful effluents to the environment, Nordic tailings fields are usually landscaped and covered with till soil from the surroundings (Hagner et al., 2021; Kauppila et al., 2013). A good tailings cover reduces oxidation and leaching of sulphide minerals in waste layers, prevents erosion, enhances vegetation restoration and increases evapotranspiration (Lottermoser, 2007). However, the sole use of forest till soil as the growth medium cover provides only low fertility and subsequently slow plant establishment (Hagner et al., 2021; Heiskanen et al., 2020).

There is a global enhancive need for reutilizing increasing amounts of organic by-products and wastes from households, industries and energy plants. Many of the recyclable waste materials, such as biochar, and composted sewage sludge have been suggested and are increasingly being used for soil improvement, landscaping and carbon sequestration to provide faster vegetation restoration on highly degraded lands (Ali et al., 2017; Méndez et al., 2015; Bolan et al., 2021). Recently these recyclable waste materials have also been tested and utilized in landscaping of mine reclamation areas (Fellet et al., 2011; Hagner et al., 2021; Page-Dumroese et al., 2018; Peltz and Harley, 2016; Trippe et al., 2021). Germination, as well as subsequent growth success, differs according to plant species and growth medium (Heiskanen et al., 2020). However, there are still few studies that focus on the feasibility of different recyclable waste materials in the cover media and their effects on plant species development in mine reclamation areas under boreal climatic conditions, where cool temperatures and a short growing season restrict vegetative growth.

The ability of various plants to grow in soils with high metal levels to take up, tolerate and bioaccumulate different elements and metals, varies considerably (Filipović-Trajković et al., 2012; Reeves and Baker, 2000). Various grasses are often used in landscaping of mine tailings due to their rapid growth and, consequently, ability to decrease erosion and retain water and nutrients (Koivuhuhta et al., 2019). Pines have been suggested for use due to their low bioaccumulation of metals (Jana et al., 2012). In addition to improved plant growth, amendments such as CaO, fly ash, organic wastes and biochar have been suggested and used to immobilize heavy metals and reduce their phytoavailability in polluted soils (Hmid et al., 2015; Mahar et al., 2016; Wang et al., 2016). Nevertheless, there are few studies on the potential of these materials for reducing the phytoaccumulation of elements and metals from mine tailings under northern conditions.

The aim of this greenhouse study was to test the properties and usability of locally available (northern Finnish) waste and side stream materials to be used in the growth layer of mine waste covers and their feasibility for vegetation restoration. One compost type (Com), three biochar types (Bc1–3) and two ash types (Ash1-2) were added to forest till soil. The physical and chemical properties of the growth media were examined as well as their effects on the germination, above- and below-ground growth and element accumulation in timothy grass (*Phleum pratense* L., trade name Uula), white clover (*Trifolium repens* L.) and Scots pine (*Pinus sylvestris* L.) seedlings during one growing period. Our earlier experiments (Hagner et al., 2021; Heiskanen et al., 2020) have shown that organic material is needed as a nitrogen source in growth media of tailings cover for vegetation restoration. Novelty of this study is that the feasibility of various biochar and ash types in growth media mixtures with compost is tested and as well as their effects on the plant growth and phytoaccumulation of metals. We hypothesised the following outcomes. Firstly, i) pure tailings or till soil alone do not enable feasible plant growth mainly due to the lack of nitrogen. Thus, sewage sludge compost was used as an organic amendment in the tailings and the glacienic till, which is commonly used as cover material. Secondly, ii) biochar added to the growth medium enhances germination and growth of plants but this response varies depending on the biochar feedstock. Thirdly, iii) mineral ash improves plant growth. Lastly, iv) biochar decreases solubility of elements and metals and thus also decrease their concentrations in plant tissues.

## 2 Materials and methods

### 2.1 Media description

Tailings soil was dug in summer 2020 from the tailings field at the Rautuvaara iron enrichment plant (Kolari, northern Finland 67°29′43.9″N, 23°55′17.3″E, with a mean yearly temperature of 0.3 °C and precipitation of 450–550 mm). The tailings soil was from the brownish, oxidized upper layer (about 20 cm deep) of the soil. The glacial till soil (acid podzol), which is commonly used in tailings covers, was dug from forest soil nearby the tailings field. The same till material was also used in the covering of the entire tailings site in Rautuvaara in 2018 and was used also in our previous experiments (Hagner et al., 2021; Heiskanen et al., 2020).

The compost used was sewage sludge composted with peat, wood chips and sand (Levin vesihuolto Oy; Sirkka, Finland; Evira acceptance code FIC009-05135/2008NA). Biochar (Bc1) was made pyrolyzing Norway spruce (*Picea abies* L. Karst) wood in a batch retort (10 m^3^) at 450 °C (by RKP Ltd., Mikkeli, Finland) as in our previous experiments (Hagner et al., 2021; Heiskanen et al., 2020). Biochars Bc2 and Bc3 were pyrolyzed (by SoilCare Oy, Mikkeli, Finland) in a small batch retort (0.8 m^3^) at 450 °C and a pyrolysis duration of 6 h Bc2 was produced from sewage sludge (from Napapiirin Energia ja Vesi Oy; Neve Oy, Rovaniemi, Finland) and pine woodchips (1:1, v v^−1^) mixed carefully before pyrolysis. Bc3 originated from waste wood chips (obtained from Pohjaset Recycling Oy, Keminmaa, Finland).

**Figure 1:**
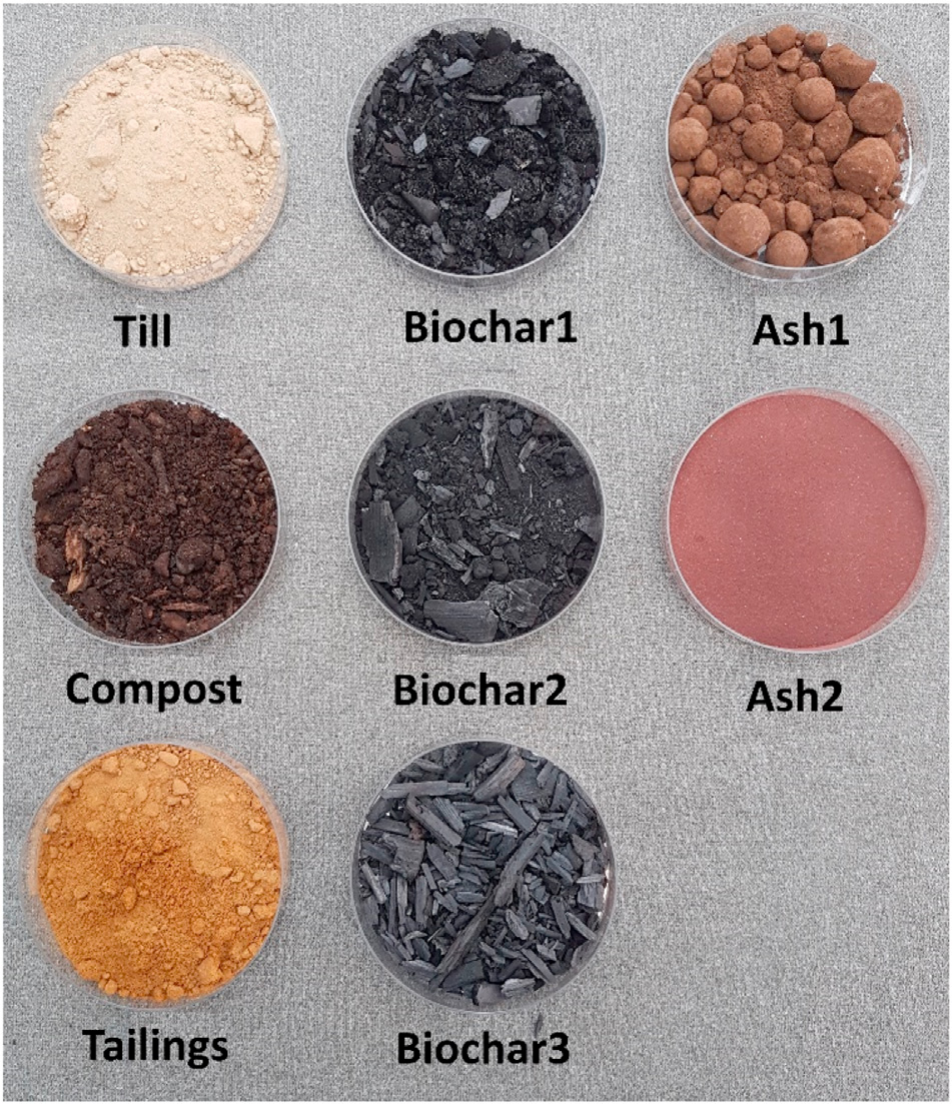
Growth media components used in the study. Petri dish diameter is 9 cm.

Ashes were obtained from Neve Oy. Ash1 was granulated fly ash from their heat plant burning wood and peat (product name: Ecolan Silva® Horus; https://www.yara.fi/lannoitus/metsa/horus/). Ash2 was produced in the pilot plant built to handle the sludge from the Rovaniemi city wastewater treatment plant by thermal treatment of sewage sludge using so termed “PAKU technology” (www.endev.fi/en/front-page). The growing media used as well as their components and mixing ratios are given in Figure 1 and Table 1.

**Table 1:** Growth media used in the growing pots (Neve Oy = Napapiirin Energia ja Vesi Oy).

Medium (12 cm thick)	Abbreviation	Volumes %	Specifier
Till	Till	100	Forest soil nearby Rautuvaara
Till + Compost	Till + Com	95 + 5	Composted sewage sludge by Levin vesihuolto Oy
Tailings	Tail	100	Upper soil layer of tailings field at Rautuvaara
Tailings + Compost	Tail + Com	95 + 5	
Till + Compost + Biohcar1	Till + Com + Bc1	90 + 5+5	Bc1 from Norway spruce by RKP Oy
Till + Compost + Biohcar2	Till + Com + Bc2	90 + 5+5	Bc2 from sewage sludge by Neve Oy and woodchips
Till + Compost + Biohcar3	Till + Com + Bc3	90 + 5+5	Bc3 from waste wood by Pohjaset Recycling Oy
Till + Compost + Ash1	Till + Com + Ash1	90 + 5+5	Ash1 is granulated fly ash by Neve Oy
Till + Compost + Ash2	Till + Com + Ash2	90 + 5+5	Ash2 is ash from sewage sludge by Neve Oy
Till + Compost + Biohcar1+Ash1	Till + Com + Bc1+Ash1	85 + 5+5 + 5	
Till + Compost + Biohcar1+Ash2	Till + Com + Bc1+Ash2	85 + 5+5 + 5	
Till + Compost + Biohcar3+Ash1	Till + Com + Bc3+Ash1	85 + 5+5 + 5	
Till + Compost + Biohcar3+Ash2	Till + Com + Bc3+Ash2	85 + 5+5 + 5	

### 2.2 Physical analyses

The particle size distribution for individual media components was measured (n = 3) using dry sieving with a series of sieves (from 20 to 0.06 mm). The bulk density (Db) of each growth medium was determined as the ratio of dry mass (dried at 105 °C) to near-saturated volume in steel cylinders (n = 3) (Dumroese et al., 2011; Heiskanen et al., 2020). The cylinders (height 60 mm, diameter 58 mm) were filled with each medium, saturated and, allowed to drain freely (to about -0.3 kPa matric potential). The particle density (Ds) was estimated using an average density of 2.65 g cm^−3^ for mineral and 1.5 g cm^−3^ for organic components. Loss-on-ignition at 550 °C for 2 h provided an approximate estimate of the organic matter for each medium (n = 3). The total porosity (TP) was estimated as ((Ds-Db)/Ds) ∗ 100.

Specific surface areas of biochars were analysed. Samples (200 mg) were pre-treated at low pressures and high temperatures to clean the surfaces. Sample tubes were immersed in liquid N (−197 °C) and N gas was added to the samples in small steps and the resulting isotherms were obtained. Specific surface areas were calculated from adsorption isotherms according to the BET method (Brunauer et al., 1938). The pore-size distribution (relative distribution of pore volumes) (vol%) was calculated from the individual volumes of micropores, mesopores, and macropores using the NLDFT (Non-Local Density Functional Theory) model.

### 2.3 Chemical analyses

Plant above and below ground parts were analysed separately. Before analysis the roots of plants were washed clean under running tap water over a 2-mm sieve. Plant and growth media samples were dried (at 60 °C for 96 h). Plant samples were homogenized with a rotor mill (Retsch ZM100 Germany, sieve of 0.75mm trapezoid holes) and growth media samples sieved (<2 mm). Total C and N of plant and growth media samples were measured (ISO 10694, 1995; ISO 13878, 1998) from sieved and dried growth media and plant samples using a Leco TruMac CN Carbon/Nitrogen Determinator (Leco Corp., St. Joseph, MI, USA). Samples for other total elements in growth media (SFS-ISO 11466, 2007) were digested by the closed wet HNO_3_–HCl digestion method in a microwave (CEM MDS-2000; CEM Corp., Matthews, NC, USA) and the extract was analysed on an iCAP 6500 Duo ICP-emission spectrometer (Thermo Scientific Ltd., Cambridge, UK). Total concentrations for elements in plant samples were analysed by the closed wet HNO_3_–H_2_O_2_ digestion method in a microwave (CEM MARS 6; CEM Corporation, USA), and iCAP 6500 Duo ICP-emission spectrometer.

Acid ammonium acetate (pH 4.65) extractant was used to assess extractable sulphur (S) (Vuorinen and Mäkitie, 1955). The quantification was done using the previously described ICP-emission spectrometer. Ammonium (NH_4_), nitrate (NO_3_), and total N in growth media were determined from a KCl-extract on a FIA-analyser (Lachat QuickChem 8000, Lachat Instruments, Milwaukee, WI, USA) (Kalra and Maynard, 1991). With a microwave (CEM MDS-2000 described above), the hot water refluxing method was used to extract easily soluble boron, which was quantified using the ICP-emission spectrometer.

For cation exchange capacity (CEC), growth media samples were prepared as for extractable nutrients (ISO 11260, 2018). We used a 0.1M BaCl_2_ solution to extract exchangeable cations, and their total concentrations in the filtrate were determined using the previously described ICP-emission spectrometer. To determine exchangeable acidity, the 0.1 M BaCl_2_ extract was titrated with a 0.05 M NaOH solution up to pH 7.8. Effective CEC [ECEC (cmol kg^−1^)] was then calculated as ECEC (in cmol kg^−1^) = Na + K + Ca + Mg + ACI_E, where ACI_E is exchangeable acidity from BaCl_2_ extract. Percentage base saturation was calculated as the sum of the bases (Na, K, Ca, Mg) divided by ECEC.

Elements in percolate water from growing pots were analysed using a modified ICP-OES-technique (ISO 11885, 2007) and nitrate and ammonium using flow analysis (SFS-EN ISO 13395, 1997; ISO 11732, 2005).

### 2.4 Plant growth experiment

The plant growth experiment was made according to a completely randomized design. Growing pots were laid randomly on steel basins (each 45 × 45 cm, 8 cm in depth), which were filled with oxidized upper tailings layer (4 cm) from the tailings field of the Rautuvaara iron enrichment plant (Kolari, northern Finland 67°29′43.9″N, 23°55′17.3″E). The basins were kept moist by spraying from above with tap water 4–5 times a week. Pots were 2 L plastic pots (A/S Bulsø Plastic, Søhus, Denmark), with open holes at the base so that plant roots could grow into the tailings soil. Prior to placing pots on the basins, pot bases were covered with a section of gauze to prevent medium from trickling through. The pots were then packed with the growth media based on till soil supplemented with 5 vol.% of compost, biochar and ash (for treatments see Figure 1, Table 1). The depth of the media in the pots was 12 cm. The pots were pressed to a depth of 10–15 mm into the tailings soil in basins. The filled pots were left to stabilize for 20 days before sowing the seeds and planting the pine seedlings.

Planting and sowing of the pots were done on 10.02.2020 (dd.mm.yyyy) in a greenhouse (Luke, Suonenjoki, Finland). Two of the three plant treatments were seeds (15 per pot) of timothy grass and white clover sown in the pots. The third treatment was one-year-old Scot pine seedlings of local central Finnish seed origin, which were planted in the pots (one per pot) with their roots washed free from peat. After 21 days from sowing, 7 white clover and 8 timothy germinants were left per pot for further growth. In the case of weak germination or poor germinant vigour, surplus germinants from the replicates of the same growth medium type were transplanted into the incomplete pots. If germination was poor in all replicates, extra seedlings from till and compost media were used as replacements. In all, there were 13 different growth media combinations with 6 replicates for 3 different plant (total of 234 pots).

Plants were grown in a greenhouse, where the natural light was supplemented with artificial diurnal light for 18 h (high pressure sodium lamps were on from 5 to 23 h). The temperature was set at 20 °C (day) and 15 °C (night). Daily temperatures at seedling shoots were 20–32 °C due to light warming. Full light yielded photosynthetically active radiation at the seedling shoot level of 380–450 μE m^−2^ s^−1^. Relative humidity ranged between 30 and 70%, being about 10% higher with no light. During the experiment, basin positions were changed monthly to reduce spatial variation in growing conditions.

Irrigation was done automatically during first two weeks by spraying pure tap water from above (for 30 s four times daily; 2–5 mm per week) and then manually 1–2 times per week using tap water supplemented with 700 nitrogen (N) and 30 μg l^−1^ phosphorus (P) (using urea and NaH_2_PO_4_) to mimic the average N and P concentrations in rainwater in northern Finland (Järvinen and Vänni, 1998; Vuorenmaa et al., 2001). No other added nutrients were used. Irrigation need was estimated visually and by weighing the pot masses from a separate extra set of pots assigned to follow the need for irrigation. Once a month, pH and electrical conductivity (EC) were monitored from two pots in each treatment and species by lifting them up from the basins and watering similarly over the target masses so that a percolated water sample of equal volume could be taken from the bottom of the pots.

Germination of the seeds was recorded at 21 days from sowing. Thereafter, plant quality and height were measured every second week. Plant shoot quality was estimated using a 5-category visual scale (0 dead, 1 dying, 2 weakened, 3 slightly symptomatic, 4 vigorous). On 04.05.2021, plants were harvested and measured for morphological attributes: height, visual vigour (greenness and chlorotic tone), and dry mass of shoot. The greenness tone was estimated using a chlorophyll content meter (CCM-300, Opti-Sciences, Hudson, New Hampshire, USA). Shoots and roots were collected, roots washed under tap water, dried at 60 °C and their dry mass weighed. Roots growing into the tailings soil in basins were not analysed because there were very few and sieving them from tailings soils would have been very difficult.

Plant samples for chemical analyses were homogenized using a sieve mill (Retsch ZM100, Retsch GmbH & Co, Haan, Germany) with 0.75 mm mesh size. In some cases the biomass was so low that the replicates of shoots and roots were pooled for each species and growth medium before milling for the plant chemical analysis. Pine needles were also divided into new (grown during experiment) and old needles (grown before planting).

### 2.5 Statistical analyses

Data were analysed with IBM SPSS Statistics (Version 27.0) and SAS (Version 9.4). One-way analysis of variance was used to compare the effect of growth medium on the plant variables (plant height and biomasses). Homogeneity of variance was tested using Levene's test. The Bonferroni method at a significance level of p < 0.05 was used for post-hoc multi-comparison tests of the differences among means of different growth media. Because of the small number of replicates (5–6), categorical variables (0–4; plant vigour and root volume) could not be analysed or tested (ordinal or binary logistic regression). However, plant mortality (dead and dying plants) among growth media was possible to test with the Fisher's exact test, but only for the extreme values (0 vs. 100%) because of the small number of replicates. Statistical analysis was not possible for element accumulation on plants because of the pooled samples in chemical analysis.

## 3 Results

### 3.1 Media physical properties

Ash2 and tailings soil were the finest media in particle size with over 50% particles smaller than 0.1 mm (Table S1). Till soil followed with about 20% of particles <0.1 mm. Compost and Ash1 were the coarsest media having 29 and 65% of particles >5 mm, respectively. Ash2 had 69% of particles <0.1 mm. Biochar types varied in their particle size distribution. Bc2 (made of sewage sludge and wood chips) had more <0.1 mm and 2–5 mm particles than those made of spruce (Bc1) and waste wood (Bc3).

Specific surface area and porosity were higher and mean pore diameters lower in biochars made from spruce wood (Bc1) and wood waste (Bc3) than those made from sewage sludge and wood ships (Bc2) (Table S2). Loss on ignition (0.56–2.61 %) and bulk density (1.11–1.80 g/cm^3^) were relatively similar in all the studied media (Table 2). Bulk density of tailings and till was reduced only slightly by mixing with additives. Total porosity was highest in tailings soil and in the mixture of tailings and compost, and lowest in till soil. Biochar and ash applications increased the total porosity and water retention of till-based growth media. Water retention at -0.3 kPa matric potential of Till + Compost was improved 3-5%-units by adding 5 vol.% biochar and/or ash.

**Table 2:** Mean loss on ignition (LOI), bulk (Db) and particle densities (Ds), total porosity (TP) and, water retention at -0.3 kPa matric potential (WC03) for the growth media (n = 3), as well as electrical conductivity (EC) and pH in water suspension (1 + 5 method) (n = 3).

Medium	LOI, %	Db, g/cm3	Ds, g/cm3	TP, vol.%	WC03, vol.%	EC, μS cm^−1^	pH
Till	0.56	1.80	2.64	31.9	27.3	9.7	7.74
Till + Compost	1.62	1.76	2.63	33.2	27.9	26.8	7.82
Tailings	1.98	1.12	2.63	57.5	49.7	1013.3	6.30
Tail + Compost	2.61	1.11	2.62	57.7	51.6	916.3	6.48
Till + Com + Bc1	1.56	1.60	2.63	39.3	32.0	38.1	7.18
Till + Com + Bc2	1.40	1.63	2.63	38.1	32.8	50.6	6.73
Till + Com + Bc3	1.11	1.64	2.64	37.8	31.3	29.8	7.04
Till + Com + Ash1	1.09	1.72	2.64	34.8	30.4	147.2	6.89
Till + Com + Ash2	1.18	1.66	2.64	37.0	32.0	69.2	6.93
Till + Com + Bc1+Ash1	1.68	1.60	2.63	39.1	31.6	370.3	6.89
Till + Com + Bc1+Ash2	1.47	1.57	2.63	40.4	34.1	332.0	7.09
Till + Com + Bc3+Ash1	2.03	1.62	2.63	38.3	31.8	82.8	7.52
Till + Com + Bc3+Ash2	1.29	1.61	2.64	38.9	31.8	63.4	7.08

### 3.2 Media chemical properties

Tailings soil contained more As, Mg, Ni and S than other media components (Table S3). Also Ca, Cr and Fe content were high in the tailings soil. The element concentrations in pure till were quite low. Compost contained substantial amounts of Al, N and P. Concentrations of Al, B, Ca, Cd, K, Mn, Na, Pb, S and Zn were highest in Ash1. Ash1 had also a high concentration of As, Cr, Fe, Mg and P. Contrastingly, Ash2 had the highest concentrations of Cr, Cu and P. Elemental concentrations in the biochars varied. Bc2 made of sewage sludge had the highest concentrations of Al, Cu, Mg, P, and, S compared with Bc1 and Bc3. The highest N content was also found in Bc2 (Table S3).

Extractable elements were lower level compared with the total concentrations in the media components (Table S4). Likewise with the total concentrations, the highest concentrations of extractable Ca, K, Mn and Na were in Ash1, where S level was also high. Tailings soil had, however, the highest S content. Ash1 had markedly higher ECEC than the other tested media components. Of the tested biochars, Bc2 had the highest total N and NH4, and Bc1 the highest Ca, K, Mg and S concentrations. Compost contained the most NO_3_ and had also a high level of extractable P, Ca, Mg, Fe and ECEC. The electrical conductivity (EC) was highest in tailings soil and the mixture of tailings and compost (Table 2). EC was lowest in till soil. pH was between 6.3 and 7.8 in all the media.

**Figure 2:**
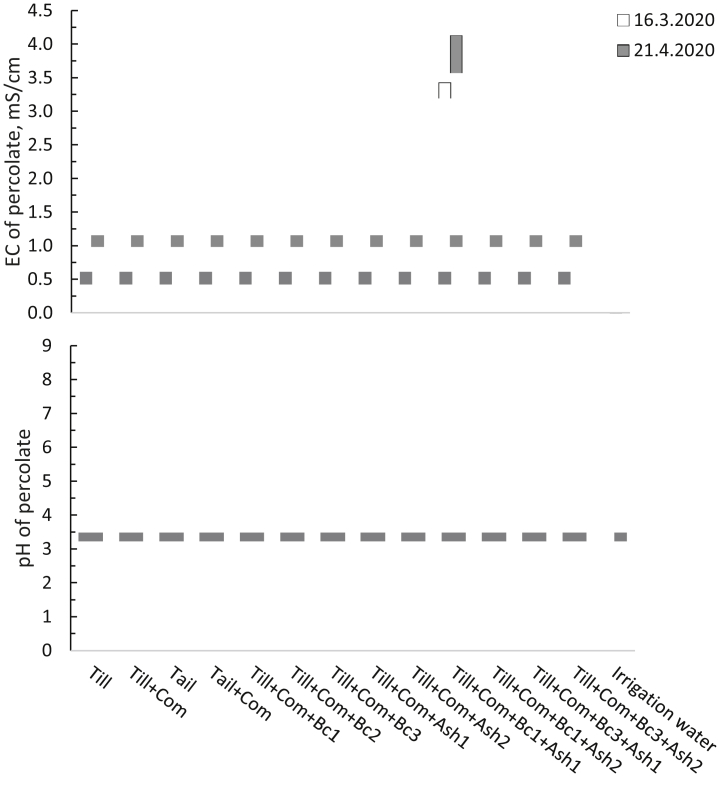
Development of acidity (pH) and electrical conductivity (EC, mS cm^−1^) from one combined percolate sample per treatment. Planting and sowing of the pots were executed on 10.02.2020 (dd.mm.yyyy). Measurements were made from a pooled percolate from 6 pots.

In the percolate water from the growing pots, pH was within the range 6.9–7.9 and EC 1.7–4.1 mS cm^−1^ during the experiment (Figure 2). During the growing period, pH tended to decrease and EC to increase. Element concentrations were relatively similar in the percolate water from the growing media pots (Table S5). NH_4_ and NO_3_ contents were highest in Till + Com + Bc2, Till + Com + Bc1+Ash1 and Till + Com + Ash, respectively. Especially N contents decreased in the percolate water during plant growth (data not shown).

### 3.3 Plant germination and growth

**Figure 3:**
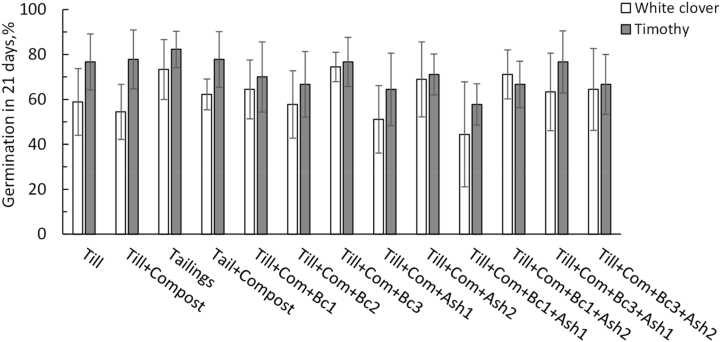
Germination success 21 days after sowing 10.2.2020 (dd.mm.yyyy) (arithmetic mean ± sd, n = 6). Differences among treatments were not statistically significant.

On average, germination success was 62.2% in white clover and 71.6% in timothy 21 days after sowing without significant differences among the treatments (Figure 3). Best germination of clover (77%) and timothy (82%) occurred in tailings soil followed by till soil, where the germination rate was slightly less in clover (59%) and timothy (78%). Compost application to till or tailings had no marked effect on the germination. Neither Bc (1,2,3) or Ash (1,2) influenced the germination. Poorest germination rate occurred in Till + Com + Bc1+Ash1 with both species.

**Figure 4:**
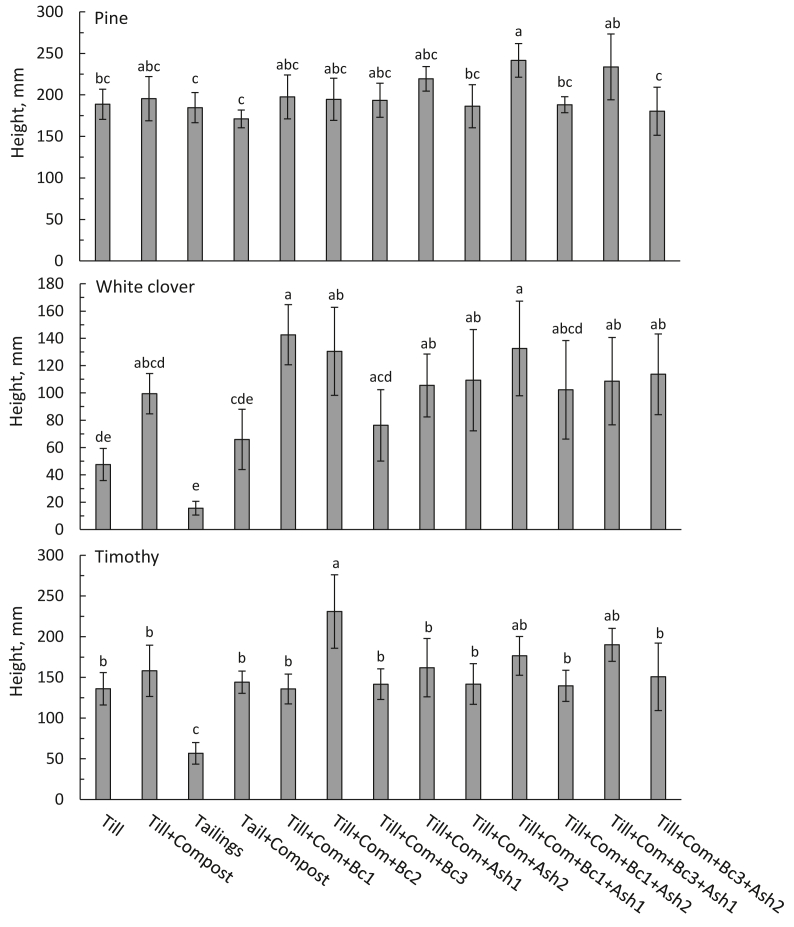
Plant height after growing in different growth media (arithmetic mean ± sd, n = 5–6). Above bars, the same letter denotes non-significant difference (p > 0.05 in Bonferroni test) among growth media.

**Figure 5:**
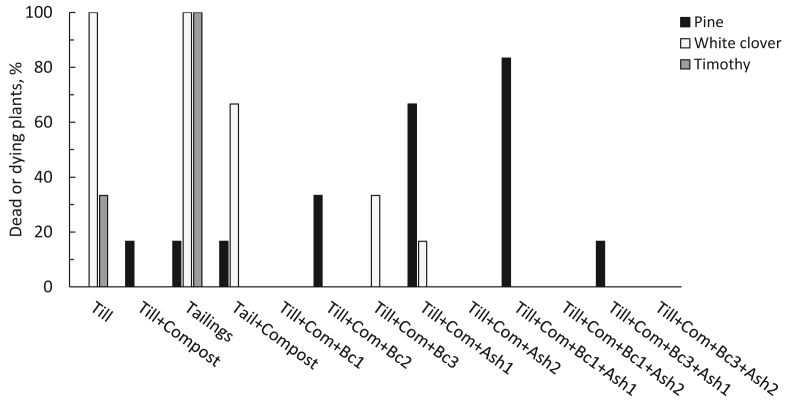
Dead and dying plants two weeks before the end of the growing experiment (%, n = 5–6).

The growth of white clover and timothy germinants declined rapidly in tailings soil, where the growth was clearly poorest (Figures 4 and 5). Compost added to tailings increased the height growth of both species (Figure 4). Clover height remained also low in pure till soil, but supplemented compost increased the final height. Interestingly, timothy grew moderately well also in pure till and compost application had no effect on its final height. Application of Bc1 and Bc2 increased clover growth further, and Bc2 also timothy growth. Added ash did not affect clover height compared with Till + Com. In general, timothy tended to grow more evenly in all growth media. The tallest timothy grew in Till + Com + Bc2.

**Figure 6:**
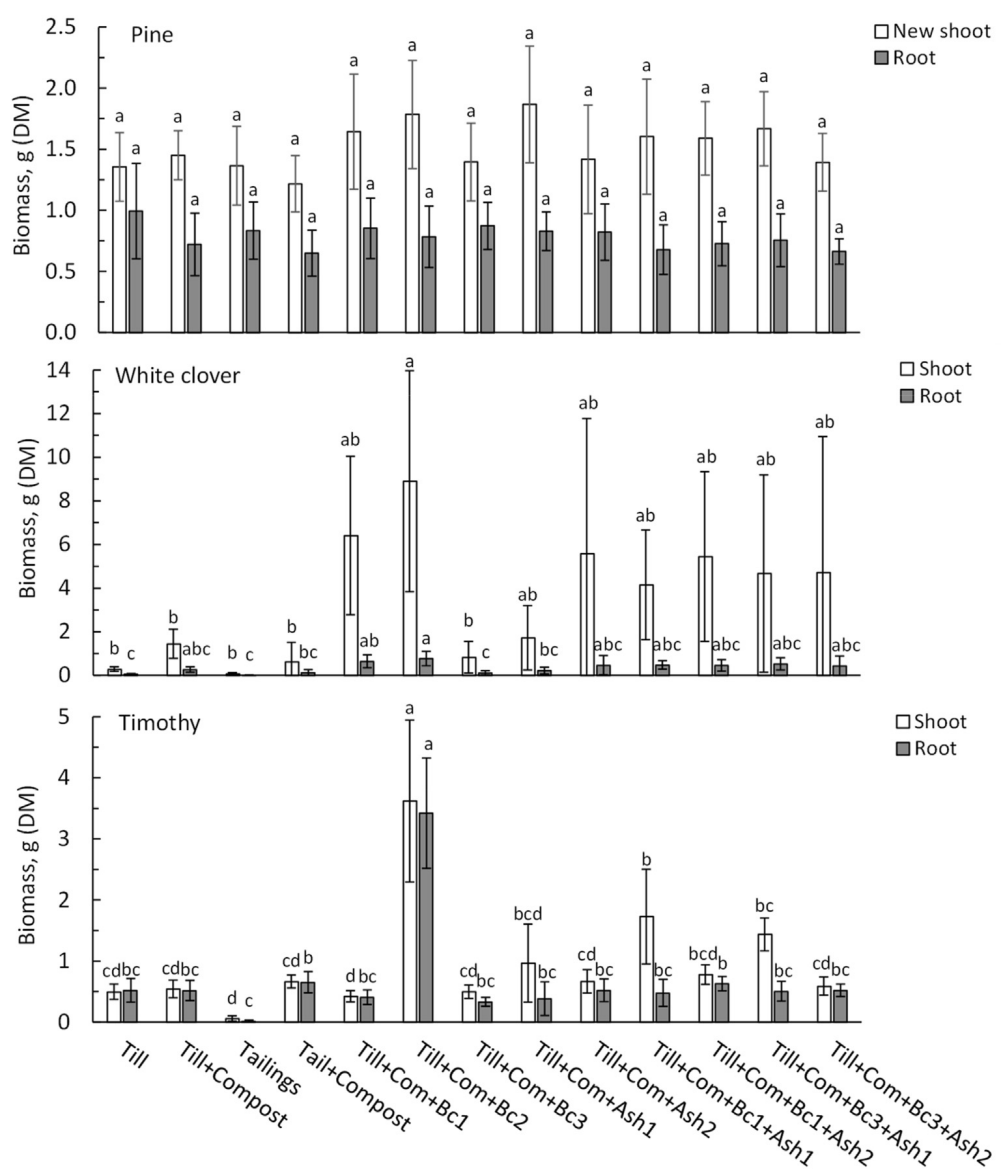
Dry plant biomass after growing in different growth media (mean ± sd, n = 5–6). Above bars of the same hue, the same letter denotes non-significant difference (p > 0.05 in Bonferroni test) among growth media.

The proportions of dead and dying white clover and timothy were highest in pure tailings and till soils (Figure 5). Both white clover and timothy had the poorest shoot and root biomass in tailings soil (Figure 6). Compost application to tailings increased the biomass of both species, but the difference was significant only for timothy roots. White clover biomass was also low in pure till but showed a slight increase with added compost. White clover shoot biomass increased markedly after application of Bc1, Bc2, Ash1, Ash2 and in their combinations. In contrast, timothy showed increased biomass only in Till + Com + Bc2 and Till + Comp + Bc1+Ash1 (Figure 6).

Pine seedlings were shortest in tailings soil with and without added compost. In addition, application of Ash2 produced the shortest seedlings (Till + Com + Ash2; Till + Com + Bc1+Ash2; Till + Com + Bc3+Ash2) although without a clear statistical significance (Figure 4). The tallest pines grew in Till + Com + Bc1+Ash1 and Till + Com + Bc3+Ash1 (Figure 4). Even Ash1 tended to promote pine height growth, later it increased seedlings mortality (Figure 5). Pine showed no significant differences in biomass among growth media (Figure 6). However, pine tended to have a somewhat larger biomass of new shoots in Till + Com + Ash1 and of roots in Till + Com + Bc1. Furthermore, till soil tended to promote the highest relative allocation to root biomass.

### 3.4 Bioaccumulation of elements

Scots pine grown in tailings soil (Tail and Tail + Com) accumulated higher concentrations of As, Al, Ca, Fe, Mg and Ni in the old needles than those grown in other growth media (Table S6). In new needles only Fe concentration was increased in Tail and Tail + Com. In addition, the roots of pines grown in tailings soil (Tail and Tail + Com) had higher concentrations of As, Cu, Mg and Ni than those grown in other media. The elements of tailings were not significantly accumulated in pine stems, except for Fe.

Added Ash1 in growth media (Till + Com + Ash1; Till + Com + Bc1+Ash1; Till + Com + Bc3+Ash1) showed increased concentration of B, Ca, K, Mg, Na and S in old pine needles as compared with Till + Com (Table S6). In new needles only concentrations of B and K increased. Application of Ash1 also increased B, Ca, Na, and S concentration in pine roots and B and Na concentration in stems, but application of Ash2 had no marked increasing effect on the concentrations of elements in needles, roots or stems.

Added biochars had no effect on the accumulation of most elements in pine needles (Table S6). However, old and new needles of pines grown in Bc1 (from spruce) and Bc2 (from sewage sludge + wood chips) media had 3-10 times lower levels of Cr and 2 times lower levels of Fe than those grown without biochar (Till + Com). Also, Bc3 reduced the concentration of Cr and Fe in new needles but only Fe in old needles. Application of Bc1 also reduced Mn concentration of pine roots, Bc1 also decreasing Al and Ni concentrations. Furthermore, stem Al and Fe levels were slightly reduced especially by Bc1 and Bc3 application. N concentration in Scots pine tissues was relatively uniform among growth media, being higher in new than in old needles. N concentration in old needles was slightly elevated in Till + Com + Bc1+Ash1. All biochar and ash applications tended to reduce Cr, Ni and Fe concentrations, especially in new needles when compared with Till + Com.

White clover and timothy did not grow roots in tailings soil. Added compost enhanced clover root growth slightly but no timothy roots grew in Tail + Com. Root development was also weak in pure till soil. In general, tissue element concentrations were relative uniform among growth media (Table S7, S8). However, added Ash1 increased B concentration in roots and shoots of timothy and white clover up to ten-fold. Also, K and P concentrations in the roots and shoots of both species increased. In contrast, Ash2 had no high effect on elemental concentrations in roots or shoots. Adding biochars had no consistent effects on element accumulation even though the wood-based biochars (Bc1, Bc3) seemed to reduce concentrations of Al, Cr, Fe Mg, Mn in roots and Cr slightly in shoots of white clover. However, Bc2 seemed to increase Al and Fe levels in shoots of timothy.

## 4 Discussion

To summarise the results of this study, the main study hypothesis was supported. This means that pure tailings or till soil alone as growth media did not promote good plant growth during the first growing season. White clover and timothy showed poorest growth on pure tailings soil, but addition of 5 vol.% of compost to the tailings and till soil increased the final height and biomass. However, planted Scots pine seedlings were unaffected by growth media. Our second assumption was that biochar addition to the till soil enhances germination and growth of herbaceous plants in particular, but their responses to various biochars differ. This assumption was partly confirmed; the biochars had no effect on the germination success of the plants. The height and biomass of white clover and timothy were improved with application of biochar produced from sewage sludge + wood chips (Bc2). White clover benefitted also from the added biochar originating from spruce wood (Bc1). Unexpectedly, added biochar produced from waste wood (Bc3) did not confer any advantage to timothy and white clover compared with till soil supplemented with compost. The biochars tested had no effect on pine growth. Our third assumption was that ash-containing minerals would improve plant growth. As expected, biomass of the timothy and white clover grown in ash containing media was higher than in media without ash, but no marked effect was evident in pine growth. Finally, we assumed that added biochar decreases solubility of elements and metals and thus also decreases their concentrations in plant tissues. This assumption was not fully confirmed, because no consistent effects of biochar on the bioaccumulation of elements was found during the first growing season, although concentrations of some metals in plant tissues were decreased.

As expected, our results showed marked differences in the properties among the studied growth media as well as in plant growth. Pure tailings soil promoted poor growth in clover and timothy. Even though the germination of both plant species was best in the moist tailings soil, their growth soon declined in it. As also found previously, planted pine seedlings were insensitive to the studied growth media and they grew relatively well also in pure tailings soil, at least for the first growing season (Hagner et al., 2021; Heiskanen et al., 2020). However, planted pine seedlings may have grown well in the first season by using their photosynthate reserves but in future seasons their growth may vary more owing to soil properties. Germination as well as the subsequent plant growth also have been found to differ by grass species and growth media (Heiskanen et al., 2020). Application of compost (5 vol.%), which contained abundant N and P, into tailings and till soil was shown here to speed up vegetation establishment as it increased the height and biomass of the herbaceous plants. This is in accordance with our previous greenhouse (Heiskanen et al., 2020) and field studies (Hagner et al., 2021). The limited rooting and growth in pure till and tailings soils were most probably due to the low soil N and increased soil density, and in the tailings soil, also to the high salinity (Charles et al., 1991; Gaheen and Njos, 1978; Hagner et al., 2021; Heiskanen et al., 2020; Krzaklewski and Pietrzykowski, 2002; Tordoff et al., 2000). Thus, organic amendments are needed for ensuring vegetation establishment in the tailings and in glacienic till, which is the most common cover material on the Nordic mine waste areas. Similar results have been reported also in other studies (Dietrich et al., 2017; Drozdowski et al., 2012; Miller and Naeth, 2017).

Contrary to our second expectation, the biochar application to the till soil did not affect germination of clover and timothy seeds, which is probably due to the adequate irrigation during germination. The possible negative effect of biochar on seed germination (Joseph et al., 2021) was avoided by using stabilization period between the placing of growth media in the pots and the sowing of seeds. However, as assumed, the further growth of these species was affected by biochars and the plant response to different biochars varied. Joseph et al. (2021) concluded also in the review that plant response to biochar application depends on biochar and soil characteristics but also plant type. They identified soil properties including texture, pH, CEC, TN, and C/N ratio as key factors that can alter the plant production response significantly. In this study, biochars produced from spruce wood (Bc1) and sewage sludge + wood chips (Bc2) increased the height and biomass growth of clover and timothy markedly. Instead, biochar made from waste wood (Bc3) did not confer any advantage compared with added compost only. In addition, none of the biochars affected pine growth. Both timothy and clover produced markedly higher biomass in the soil supplemented with biochar produced from sewage sludge. Sewage sludge biochar had the smallest particle surface area and porosity, as shown also by Leng et al. (2021), but undoubtedly these attributes were less important for water retention and availability in this experiment where the plants were irrigated. The main differences among the tested biochars were in the total N, P and soluble N (especially NH_4_) concentrations, which were several times higher in the sewage sludge biochar. Wood-based biochar added to soil might immobilize N so that N was less available to plants (Clough et al., 2013; Nguyen et al., 2017; Prommer et al., 2014), which may explain why timothy grew more poorly than in soil supplemented with other Bc-types. On the other hand, biochars added to soil may increase N net mineralization and NH_4_ concentration (Gundale et al., 2016). Because clover can fix atmospheric N by root bacteria, it could grow well also in biochar made of spruce wood. There were no marked differences in the concentrations of total and extractable elements or physical attributes of biochars made of spruce (Bc1) and waste wood (Bc3). Neither the quality of percolate water from the growth media including Bc 1 and Bc3, nor the elemental content of plants grown in media containing Bc1 and Bc3 varied substantially. The reason for the weaker growth in growth media containing waste-wood biochar remains unclear. One reason might be the lower total N concentration and higher N/C ratio of wood waste biochar compared to spruce and sewage sludge originating biochars as Dai et al. (2020) connected these attributes of biochar to lower plant productivity. However, as waste wood biochar had no major negative effect on plant survival or growth, its utilization on mining sites may still be worthwhile for example from the perspective of C sequestration.

As we expected, regarding the third hypothesis, also application of fly ash (Ash1) and ash originating from sewage sludge (Ash2) improved height and biomass growth of clover. Various wood and peat fly ashes are being used as forest fertilizers which replace the nutrients removed in biomass harvesting, counteract soil acidification and improve tree growth (Budhathoki and Väisänen, 2016). Fly ash contains essential elements like P, Ca and K but also elements such as B and metals that can be toxic to the plants (Panda and Biswal, 2018). In our study, no clear effect on timothy height or biomass was evident, although when adding spruce- or waste-wood biochar and fly ash, the above-ground biomass of timothy increased. Ashes had no marked effect on pine growth either, even though the fly ash tended (Ash1) to promote pine height. However, fly ash also seemed to increase mortality later, which the high availability and accumulation of nutrients (especially B, Ca, Mg, P, S, Zn) in Ash1-containing media probably contributed to. Increased mortality have also been reported to be associated with high element contents and CEC (Panda and Biswal, 2018). Thus, application dose of this kind of fly ash should probably be lower than that used in this experiment. On the other hand, sewage sludge originating ash (Ash2) has lower concentrations of elements with the exception of P, and thus could likely be used in higher volumes depending on P need of plants.

The bioaccumulation of elements was not consistently affected by the biochar added to the growth medium. The concentrations of some metals in plant tissues were decreased but the effect seemed to vary according to added biochar, plant species and tissue partition. In general, tissue element concentrations were relatively uniform and at common levels among growth media (Hagner et al., 2021). The As concentration was, however, relatively high in the pure tailings soil (104 mg kg^−1^) and in pure Ash1 (52 mg kg^−1^); the natural levels of As in soil usually range from 1 to 40 mg kg^−1^, with a mean of 5 mg kg^−1^ (Chou and Harper, 2007). In Finland, the largest ecologically allowable soil As concentration is 56 mg kg^−1^ and on industrial areas 250 mg kg^−1^ (Reinikainen, 2007). Nevertheless, As was here only moderately accumulated into the tissues of roots and old needles of Scots pine in media containing tailings soil. In those growth media, also timothy had elevated As levels in roots but not in shoot. The contamination by tailings particles attached to root samples (despite roots having been washed) could contribute to the elevated As level (Heiskanen and Rikala, 2000). As shown previously by Hagner et al. (2021) on the Rautuvaara tailings field, the concentrations of heavy metals (Al, Cr, Cu, Ni, Pb, As) are mostly accumulated in root tissues (even 5–40 times more) rather than in shoots. Therefore, it is probable that no harmful levels of As or other heavy metals from the tailings soil raise levels in the growth medium or accumulate in plant shoots. Thus, no harmful levels are expected to occur in their detritus. According to Joseph et al. (2021), biochar application to soil can decrease plant tissue concentrations of heavy metals by 17%–39%. In this study, biochar added to the growth medium did not have a clear effect on the bioaccumulation of elements during the first growing season. However, biochar added to growth media may later decrease the plant's uptake of heavy metals as was recently concluded by Natasha et al. (2021).

## 5 Conclusions

This study was conducted to supplement the few studies that have investigated the feasibility of using recyclable waste materials as a growth medium layer for the cover systems of mine tailings and their effects on vegetation restoration and element bioaccumulation under the northern conditions. Forest till soil is the most commonly used cover material on boreal mine tailings. However, our previous studies (Hagner et al., 2021; Heiskanen et al., 2020) showed that till media without a nitrogen-containing organic additive can promote relatively poor growth mainly due to the lack of nitrogen and partly because of high soil density. In this study, the added compost improved vegetative growth in tailings soil and in till soil. Suitable types of biochar and ash amendments showed further increase in plant growth. In addition, biochar and ash amendments are beneficial when also considering their possible life-cycle costs and longer-term effects on soil fertility, plant growth and carbon sequestration. Our results also suggest that Scots pine seedlings can thrive on various growth medium covers on tailings at least for the first growing season. Additionally, herbaceous plants (as timothy and white clover here) can grow well on the amended till media. Timothy showed faster establishment and growth than white clover. However, white clover is a recommended species in the seed mix due to its capacity to fix nitrogen in the soil from the atmosphere which in turn helps vegetation growth and recovery later. In addition, clover seems to benefit more from biochar application than timothy or pine. The long-term effects of the chemical and physical properties of cover media of mine tailings on the fertility, phytotoxicity, element accumulation in plants and on plant growth on mine reclamation sites should be assessed in further studies. Further research is also needed to demonstrate the potential of recyclable waste materials to promote circular economy, decrease environmental footprint of mining industry and contribute to C sequestration.

## Declarations

### Author contribution statement

Juha Heiskanen: Conceived and designed the experiments; Performed the experiments; Analyzed and interpreted the data; Contributed reagents, materials, analysis tools or data; Wrote the paper.

Hanna Ruhanen: Performed the experiments; Contributed reagents, materials, analysis tools or data.

Marleena Hagner: Conceived and designed the experiments; Wrote the paper.

### Funding statement

This work was supported by European Regional Development Fund (A76452).

### Data availability statement

Data included in article/supplementary material/referenced in article.

### Declaration of interests statement

The authors declare no conflict of interest.

### Additional information

No additional information is available for this paper.

## Appendix A Supplementary data

Heliyon_Supplementary_material_Heiskanen_et_al Heliyon_Supplementary_material_Heiskanen_et_al
