# Supplementary material for: Effects of compost, biochar and ash mixed in till soil cover of mine tailings on plant growth and bioaccumulation of elements: A growing test in a greenhouse
Source: Heliyon. 2022 Jan 27;8(2):e08838. doi: 10.1016/j.heliyon.2022.e08838 (PMC8816668; doi:10.1016/j.heliyon.2022.e08838)
Supplement: Heliyon_Supplementary_material_Heiskanen_et_al [file mmc1.docx]

**Supplementary Material**

**Supplementary Table 1.** Mean particle size distribution of the growth media components (proportions in DM percentages from dry sieving; n=3).

|  | <0.1 mm | 0.1-1 mm | 1-2 mm | 2-5 mm | 5-10 mm | >10 mm |
| --- | --- | --- | --- | --- | --- | --- |
| Till | 20.55 | 47.35 | 14.24 | 9.06 | 4.68 | 4.11 |
| Tailings | 52.76 | 42.90 | 3.34 | 0.83 | 0.16 | 0.02 |
| Compost | 2.47 | 27.73 | 18.11 | 22.55 | 17.27 | 11.87 |
| Bc1 | 0.04 | 21.51 | 20.42 | 28.67 | 26.63 | 2.73 |
| Bc2 | 6.79 | 18.39 | 17.58 | 42.70 | 11.71 | 2.84 |
| Bc3 | 4.91 | 27.25 | 28.19 | 27.78 | 9.56 | 2.33 |
| Ash1 | 1.10 | 11.26 | 4.22 | 18.13 | 64.90 | 0.40 |
| Ash2 | 68.70 | 30.34 | 0.53 | 0.31 | 0.07 | 0.04 |

**Supplementary Table 2.** Specific surface area and porosity estimates for the biochars. Abbreviations: BET = Brunauer-Emmett-Teller, NLDTF = Non-Local Density Functional Theory (analyses by University of Oulu Research Unit of Applied Chemistry). Biochar feedstock: Bc1 = spruce wood; Bc2 = sewage sludge + woodchips; Bc3 = waste wood.

| Quantity | Unit | Bc1 | Bc2 | Bc3 |
| --- | --- | --- | --- | --- |
| BET-model |  |  |  |  |
| specific surface area | cm^2^ g^-1^ | 180 | 2.5 | 137 |
| NLDFT-model |  |  |  |  |
| total pore volume | cm^3^ g^-1^ | 0.0673 | 0.0066 | 0.0587 |
| micropores (<2.2 nm) | cm^3^ g^-1^ | 0.0610 | 0.0005 | 0.0485 |
| mesopores (2.2-50 nm) | cm^3^ g^-1^ | 0.0060 | 0.0050 | 0.0095 |
| macropores (50 > nm) | cm^3^ g^-1^ | 0.0010 | 0.0011 | 0.0007 |
| micropores (<2.2 nm) | % | 91 | 8 | 83 |
| mesopores (2.2-50 nm) | % | 8 | 72 | 16 |
| macropores (50 > nm) | % | 1 | 20 | 1 |

**Supplementary Table 3.** Total element concentrations of the growth media components (from one combined sample, extractant aqua regia: HNO_3_+HCl). Feedstocks: Bc1 = spruce wood; Bc2 = sewage sludge + woodchips; Bc3 = waste wood, Ash1 = fly ash (peat, wood), Ash2 = sewage sludge. Highest values are in bold to highlight the differences among the media.

| Element | Unit | Till | Tailings | Compost | Bc1 | Bc2 | Bc3 | Ash1 | Ash2 |
| --- | --- | --- | --- | --- | --- | --- | --- | --- | --- |
| Al | mg/kg | 7 570 | 10 700 | **23 000** | 652 | 4 260 | 315 | **27 800** | 8 990 |
| As | mg/kg | <1.01 | **104** | 8.1 | <2.08 | <2.09 | 10 | **52** | <1.03 |
| B | mg/kg | 0.50 | 2.4 | 82 | 20 | 3.3 | 5.9 | **388** | 3.0 |
| Ca | mg/kg | 5 480 | **44 100** | 10 100 | 15 000 | 15 300 | 2 240 | **98 600** | 17 600 |
| Cd | mg/kg | 0.08 | 1.9 | 0.43 | 1.83 | 0.67 | 0.18 | **4.24** | 0.47 |
| Cr | mg/kg | 25 | **96** | 29 | 2.8 | 32 | 12 | **85** | **97** |
| Cu | mg/kg | 13 | 93 | 135 | 10 | **305** | 27 | 187 | **311** |
| Fe | mg/kg | 18 100 | **67 100** | 29 700 | 1 920 | 158 000 | 2 460 | **84 600** | 172 000 |
| K | mg/kg | 1 320 | 2 960 | 1 310 | 3 360 | 2 880 | 960 | **20 600** | 2 080 |
| Mg | mg/kg | 3 050 | **24 400** | 3 250 | 1 340 | 3 000 | 335 | **15 200** | 3 930 |
| Mn | mg/kg | 194 | 695 | 412 | 916 | 526 | 103 | **3 180** | 472 |
| Na | mg/kg | 373 | 173 | 376 | 98 | 469 | 352 | **4 580** | 2 100 |
| Ni | mg/kg | 10 | **313** | 17 | 3.7 | 29 | 2.5 | 47 | 58 |
| P | mg/kg | 602 | 367 | 11 500 | 777 | **34 000** | 427 | 18 300 | **35 600** |
| Pb | mg/kg | 1.7 | 2.6 | 7.2 | <2.08 | 15 | 4.7 | **81** | 14 |
| S | mg/kg | 76 | **16 800** | 3 000 | 439 | 2 860 | 111 | **14 800** | 362 |
| Zn | mg/kg | 16 | 16 | 192 | 228 | 695 | 124 | **1 080** | **773** |
| C | % DM | 0.2 | 1.9 | 22 | **72** | 46 | **86** | 0.64 | <0.10 |
| N | % DM | <0.08 | <0.08 | **1.4** | 0.82 | **2.3** | 0.63 | <0.08 | <0.08 |
| C/N | - | >2.5 | >23 | 16 | **88** | 20 | **137** | >8.0 | - |

**Supplementary Table 4.** Extractable nutrients of the growth media components (from one combined sample, extractant BaCl_2_). ECEC denotes effective cation exchange capacity and BS base saturation. *) Extractant AAc pH 4.65, **) Extractant KCl. Feedstocks: Bc1 = spruce wood; Bc2 = sewage sludge + woodchips; Bc3 = waste wood, Ash1 = fly ash (peat, wood), Ash2 = sewage sludge. Highest values are in bold to highlight the differences among the media.

| Element | Unit | Till | Tailings | Compost | Bc1 | Bc2 | Bc3 | Ash1 | Ash2 |
| --- | --- | --- | --- | --- | --- | --- | --- | --- | --- |
| Al | mg/kg | 2.2 | <0.244 | **37** | <1.10 | <0.898 | <1.37 | <0.257 | 0.87 |
| Ca | mg/kg | 240 | 1 910 | 4 660 | 2 000 | 202 | 372 | **11 900** | 321 |
| Fe | mg/kg | 0.17 | 0.069 | **31** | <0.219 | 3.9 | <0.275 | <0.0514 | 0.33 |
| K | mg/kg | 18.3 | 55.7 | 528 | **1 100** | 76 | 485 | **8 830** | 69 |
| Mg | mg/kg | 45.7 | 266 | **546** | 416 | 36 | 40 | **678** | 35 |
| Mn | mg/kg | 3.1 | 0.81 | **57** | 23 | 2.6 | 13 | 0.19 | 1.2 |
| Na | mg/kg | 3.6 | 8.5 | 66 | 15 | 10 | 126 | **1 450** | 56 |
| P | mg/kg | <0.305 | <0.407 | **5.1** | **5.0** | **4.8** | <2.29 | 1.08 | 0.588 |
| S *) | mg/kg | 3.6 | **9 020** | 674 | 168 | 62 | 28 | **8 050** | 313 |
| N_tot_ **) | mg/kg | 6.4 | 6.8 | **228** | 17 | **390** | 6.8 | 136 | 1.9 |
| NO_3_ **) | mg/kg | <0.499 | <0.509 | **133** | 5.9 | <0.513 | <0.51 | 2.4 | <0.502 |
| NH_4_ **) | mg/kg | 5.3 | 3.3 | 23 | 2.2 | **253** | 4.7 | **101** | 1.1 |
| ECEC | cmol/kg | 1.6 | 12 | 30 | 16 | 1.5 | 4.0 | **94** | 2.3 |
| BS | % | 100 | 100 | 97 | 100 | 100 | 100 | 100 | 100 |

| Element | Till | Till+Compost | Tailings | Tail+Compost | Till+Com+Bc1 | Till+Com+Bc2 | Till+Com+Bc3 | Till+Com+Ash1 | Till+Com+Ash2 | Till+Com+Bc1+Ash1 | Till+Com+Bc1+Ash2 | Till+Com+Bc3+Ash1 | Till+Com+Bc3+Ash2 | Irrigation water |
| --- | --- | --- | --- | --- | --- | --- | --- | --- | --- | --- | --- | --- | --- | --- |
| As | <0.010 | <0.010 | <0.010 | <0.010 | <0.010 | <0.010 | <0.010 | <0.010 | <0.010 | <0.010 | <0.010 | <0.010 | 0.0101 | <0.010 |
| Al | <0.005 | <0.005 | <0.005 | <0.005 | <0.005 | <0.005 | <0.005 | <0.005 | <0.005 | <0.005 | <0.005 | <0.005 | <0.005 | <0.005 |
| B | 0.023 | 0.143 | 0.056 | 0.103 | 0.111 | 0.111 | 0.152 | 3.39 | 0.15 | **5.21** | 0.254 | **5.91** | 0.128 | 0.0041 |
| Ca | 474 | 425 | 539 | **565** | 403 | 372 | 343 | 496 | 469 | **567** | 474 | 485 | 396 | 14.6 |
| Cd | 0.0007 | <0.0007 | <0.0007 | <0.0007 | <0.0007 | <0.0007 | <0.0007 | <0.0007 | <0.0007 | <0.0007 | <0.0007 | <0.0007 | <0.0007 | <0.0007 |
| Cr | <0.001 | <0.001 | <0.001 | <0.001 | <0.001 | <0.001 | <0.001 | <0.001 | <0.001 | <0.001 | <0.001 | <0.001 | <0.001 | <0.001 |
| Cu | 0.0037 | 0.0073 | 0.0024 | 0.0042 | 0.0071 | 0.0114 | 0.0068 | 0.0113 | 0.0111 | 0.0073 | **0.0116** | 0.0089 | 0.0084 | 0.0096 |
| Fe | 0.0044 | 0.0033 | <0.001 | <0.001 | 0.001 | 0.014 | 0.0049 | **0.026** | 0.017 | 0.0015 | 0.0087 | 0.0042 | 0.0028 | <0.001 |
| K | 42.6 | 29.1 | 68.9 | 70.3 | 37.1 | 33.3 | 23.7 | 125 | 34.5 | **169** | 41.2 | **202** | 36.5 | 1.41 |
| Mg | **275** | 203 | **359** | 265 | 162 | 187 | 87.7 | 195 | 209 | 255 | 229 | 160 | 210 | 1.53 |
| Mn | 1.61 | 3.38 | 0.099 | 0.418 | 0.797 | 3.55 | 1.74 | 3.83 | **4.1** | 1.46 | 3.38 | 1.04 | 2.66 | 0.0009 |
| Na | 29.7 | 21.2 | 29.1 | 29 | 22.6 | 22.8 | 17.0 | 54.9 | 25.4 | **84.4** | 27.7 | **72.7** | 28.1 | 2.37 |
| Ni | 0.0265 | 0.0281 | 0.0202 | 0.0354 | 0.0162 | 0.0313 | 0.0172 | 0.0232 | **0.0359** | 0.0111 | 0.0322 | 0.0091 | 0.0248 | <0.002 |
| P | 0.0146 | 0.0199 | 0.0199 | 0.0481 | 0.0171 | 0.0212 | 0.0125 | 0.0156 | 0.119 | 0.0902 | **0.180** | 0.0655 | 0.159 | 0.0282 |
| Pb | <0.005 | <0.005 | <0.005 | <0.005 | <0.005 | <0.005 | <0.005 | <0.005 | <0.005 | <0.005 | <0.005 | <0.005 | <0.005 | <0.005 |
| S | 724 | 593 | **894** | 776 | 511 | 514 | 380 | 635 | 631 | 696 | 659 | 604 | 582 | 3.34 |
| Si | 4.68 | 6.30 | 0.821 | 1.42 | 4.60 | 7.18 | 7.99 | 6.76 | **9.14** | 5.18 | 8.11 | 5.50 | 7.89 | 6.20 |
| Zn | 0.059 | 0.0557 | 0.0247 | 0.0143 | 0.0359 | 0.0326 | 0.0239 | **0.0778** | 0.0475 | 0.012 | 0.0315 | 0.016 | 0.0262 | 0.0168 |
| NH_4_ | 0.109 | 0.484 | 0.152 | 0.115 | 0.176 | **2.38** | 0.342 | 1.30 | 0.548 | 1.45 | 0.382 | 0.716 | 0.199 | <0.03 |
| NO_2_+NO_3_ | 0.83 | 1.38 | 1.27 | 0.37 | 0.905 | **2.28** | 0.236 | 1.55 | 0.853 | **2.71** | 1.22 | 1.88 | 0.257 | 0.076 |

**Supplementary Table 5.** Chemical analysis of percolate water taken from growing pots at 16.3.2020 (combined sample per treatment). Feedstocks: Bc1 = spruce wood; Bc2 = sewage sludge + woodchips; Bc3 = waste wood, Ash1 = fly ash (peat, wood), Ash2 = sewage sludge. Highest values are in bold to highlight the differences among the media.

**Supplementary Table 6.** Total element concentrations (mg kg^-1^) of Scots pine seedlings at harvest (from one combined sample. extractant: HNO_3_+H_2_O_2_). Highest values are in bold to highlight the differences among the media.

| **Compartment** | **Medium** | **C** | **N** | **As** | **Al** | **B** | **Ca** | **Cd** | **Cr** | **Cu** | **Fe** | **K** | **Mg** | **Mn** | **Na** | **Ni** | **P** | **Pb** | **S** | **Zn** |
| --- | --- | --- | --- | --- | --- | --- | --- | --- | --- | --- | --- | --- | --- | --- | --- | --- | --- | --- | --- | --- |
| New needles | Till | 48 | 0.5 | <1.52 | **94.8** | 15 | 3610 | <0.1 | 0.638 | **3.66** | 76.9 | 7510 | 1730 | **423** | 106 | 0.775 | 984 | <1.52 | 2080 | **30** |
| New needles | Till+Com | 48 | 0.5 | <1.53 | 69.5 | 27 | 2820 | <0.1 | **2.72** | 2.72 | 84.0 | 7510 | 1680 | 306 | 83.8 | **2.40** | 994 | <1.53 | 1860 | 28 |
| New needles | Tail | 48 | 0.6 | <1.53 | 57.8 | 12 | 3680 | <0.1 | 0.932 | 2.83 | **218** | 6210 | **3180** | 163 | 102 | 1.79 | 828 | <1.53 | **4010** | 20 |
| New needles | Tail+Com | 48 | 0.5 | <1.53 | 42.4 | 13 | 2970 | <0.1 | 0.519 | 2.01 | **136** | 5990 | 2490 | 111 | 48.9 | 1.31 | 839 | <1.53 | 3080 | 16 |
| New needles | Till+Com+Bc1 | 48 | 0.6 | <1.52 | 40.4 | 29 | 3000 | <0.1 | <0.305 | 2.19 | 41.8 | 7680 | 1970 | 265 | 58.7 | 0.548 | 994 | <1.52 | 2060 | 27 |
| New needles | Till+Com+Bc2 | 48 | 0.6 | <1.55 | 42.2 | 19 | 2710 | <0.1 | 0.881 | 2.21 | 42.2 | 6330 | 1580 | 317 | 50.7 | 1.42 | 992 | <1.55 | 2050 | 24 |
| New needles | Till+Com+Bc3 | 48 | 0.5 | <1.51 | 79.0 | 27 | 3200 | <0.1 | 0.363 | 3.07 | 42.5 | 7480 | 1590 | 326 | 77.2 | 0.711 | 1050 | <1.51 | 1860 | 27 |
| New needles | Till+Com+Ash1 | 48 | 0.6 | <1.52 | 43.2 | **318** | 3200 | <0.1 | 0.532 | 1.90 | 45.7 | **11000** | 2100 | 257 | 117 | 0.593 | 1020 | <1.52 | 2170 | 18 |
| New needles | Till+Com+Ash2 | 48 | 0.5 | <1.53 | 66.4 | 28 | 3310 | <0.1 | 0.473 | 3.02 | 67.5 | 7770 | 1790 | 405 | 106 | 0.733 | 1120 | <1.53 | 2310 | 28 |
| New needles | Till+Com+Bc1+Ash1 | 47 | **0.8** | <1.52 | 26.3 | **377** | **4020** | <0.1 | 0.319 | 2.78 | 45.8 | **16500** | **3000** | 150 | **712** | 0.471 | **1230** | <1.52 | 2680 | 22 |
| New needles | Till+Com+Bc1+Ash2 | 48 | 0.5 | <1.53 | 42.7 | 32 | 3000 | <0.1 | <0.306 | 3.12 | 81.6 | 7740 | 1730 | 277 | 112 | 0.489 | 1080 | <1.53 | 2280 | 25 |
| New needles | Till+Com+Bc3+Ash1 | 47 | 0.6 | <1.52 | 25.5 | **301** | 2850 | <0.1 | <0.305 | 2.32 | 52.5 | **13400** | 1720 | 161 | 233 | 0.442 | 1060 | <1.52 | 2580 | 18 |
| New needles | Till+Com+Bc3+Ash2 | 48 | 0.5 | <1.52 | 49.8 | 24 | 2940 | <0.1 | 0.987 | 2.64 | 49.9 | 7840 | 2110 | 335 | 88.8 | 0.622 | 1190 | <1.52 | 2610 | 27 |
| Old needles | Till | 47 | 0.4 | <1.53 | 293 | 17 | 5560 | <0.1 | 1.30 | 6.66 | 476 | 4110 | 2760 | 477 | 656 | 1.61 | 606 | <1.53 | 3900 | 45 |
| Old needles | Till+Com | 47 | 0.4 | <1.53 | 242 | 43 | 4330 | <0.1 | 1.87 | 4.88 | 337 | 4380 | 2230 | 425 | 466 | 1.03 | 563 | <1.53 | 2880 | 45 |
| Old needles | Tail | 46 | 0.5 | **4.63** | **312** | 21 | **10800** | 0.123 | **3.24** | **8.66** | **1630** | 5460 | 5990 | 318 | 616 | **8.63** | 553 | <1.54 | **11700** | 43 |
| Old needles | Tail+Com | 46 | 0.5 | **3.97** | **300** | 23 | 9110 | <0.1 | **2.96** | 7.10 | **1620** | 4730 | 4560 | 311 | 406 | **8.80** | 574 | <1.54 | 8900 | 36 |
| Old needles | Till+Com+Bc1 | 48 | 0.4 | <1.55 | 163 | 45 | 5810 | 0.124 | 0.881 | 4.25 | 237 | 4860 | 3110 | 367 | 565 | 1.00 | 631 | <1.55 | 4430 | 47 |
| Old needles | Till+Com+Bc2 | 48 | 0.4 | <1.55 | 186 | 38 | 5240 | 0.124 | 0.976 | 4.96 | 279 | 3990 | 2010 | 510 | 377 | 0.883 | 567 | <1.55 | 2870 | 59 |
| Old needles | Till+Com+Bc3 | **49** | 0.4 | <1.56 | 107 | 38 | 4470 | <0.1 | 1.80 | 5.85 | 127 | 4470 | 1400 | 345 | 307 | 0.876 | 658 | <1.56 | 1920 | 43 |
| Old needles | Till+Com+Ash1 | 46 | 0.6 | <1.56 | 265 | **648** | 9240 | 0.14 | 1.06 | 5.97 | 481 | 7730 | **6280** | **606** | **1820** | 1.20 | 699 | <1.56 | 7770 | **64** |
| Old needles | Till+Com+Ash2 | **49** | 0.5 | <1.56 | 168 | 43 | 5050 | <0.1 | 0.939 | 6.45 | 255 | 4770 | 1720 | 408 | 427 | 1.06 | 716 | <1.56 | 2930 | 43 |
| Old needles | Till+Com+Bc1+Ash1 | 46 | **1.0** | <1.56 | 265 | **634** | **11700** | 0.172 | 1.77 | 7.35 | 467 | **11200** | **7010** | 420 | **2390** | 1.19 | **994** | <1.56 | 7520 | **72** |
| Old needles | Till+Com+Bc1+Ash2 | 47 | 0.5 | <1.54 | 134 | 49 | 5590 | 0.107 | 0.629 | 5.37 | 259 | 4660 | 1890 | 349 | 533 | 0.814 | 664 | <1.54 | 3800 | 37 |
| Old needles | Till+Com+Bc3+Ash1 | 46 | 0.6 | <1.54 | 243 | **591** | **10100** | 0.138 | 1.18 | 6.92 | 426 | **10700** | 4300 | 391 | **1750** | 0.984 | 752 | <1.54 | **8950** | 53 |
| Old needles | Till+Com+Bc3+Ash2 | 47 | 0.5 | <1.54 | 172 | 38 | 5240 | <0.1 | 0.922 | 4.56 | 313 | 4440 | 2570 | 450 | 508 | 1.03 | 704 | <1.54 | 4170 | 48 |

**Supplementary Table 6 continued.** Total element concentrations (mg kg^-1^) of Scots pine seedlings at harvest (from one combined sample. extractant: HNO_3_+H_2_O_2_). Highest values are in bold to highlight the differences among the media.

| **Compartment** | **Medium** | **C** | **N** | **As** | **Al** | **B** | **Ca** | **Cd** | **Cr** | **Cu** | **Fe** | **K** | **Mg** | **Mn** | **Na** | **Ni** | **P** | **Pb** | **S** | **Zn** |
| --- | --- | --- | --- | --- | --- | --- | --- | --- | --- | --- | --- | --- | --- | --- | --- | --- | --- | --- | --- | --- |
| Stem | Till | 50 | 0.3 | <1.54 | **114** | 11 | 3670 | <0.1 | 0.860 | **3.33** | 116 | 4150 | 1460 | **221** | 363 | 0.829 | 783 | <1.54 | 2130 | 27 |
| Stem | Till+Com | 50 | 0.3 | <1.52 | 75.6 | 13 | 2690 | <0.1 | 0.350 | 2.92 | 82.0 | 4130 | 1500 | **193** | 420 | 0.593 | 734 | <1.52 | 1870 | 28 |
| Stem | Tail | 50 | 0.3 | <1.54 | 62.6 | 11 | 4090 | <0.1 | 0.492 | 2.78 | **251** | 4070 | **2400** | 70 | 306 | **1.77** | 555 | <1.54 | **3970** | 22 |
| Stem | Tail+Com | 50 | 0.3 | <1.53 | 49.6 | 11 | 3940 | <0.1 | **0.901** | 2.66 | **198** | 4080 | 1940 | 66 | 248 | **1.53** | 645 | <1.53 | 3210 | 22 |
| Stem | Till+Com+Bc1 | 50 | 0.3 | <1.54 | 57.0 | 14 | **4550** | 0.108 | 0.308 | 2.84 | 68.1 | 4510 | 1880 | 129 | 531 | 0.447 | 734 | <1.54 | 3340 | 26 |
| Stem | Till+Com+Bc2 | 50 | 0.4 | <1.52 | 71.1 | 13 | 3450 | <0.1 | 0.335 | 2.82 | 72.8 | 4120 | 1330 | 177 | 234 | 0.488 | 808 | <1.52 | 1970 | **31** |
| Stem | Till+Com+Bc3 | 50 | 0.3 | <1.54 | 64.5 | 13 | 3300 | <0.1 | <0.309 | 3.01 | 42.6 | 4410 | 1060 | 142 | 269 | 0.401 | 758 | <1.54 | 1450 | 25 |
| Stem | Till+Com+Ash1 | 50 | 0.4 | <1.50 | 48.7 | **45** | 3890 | <0.1 | <0.300 | 2.64 | 56.9 | 4950 | 1820 | 122 | 1670 | 0.450 | 613 | <1.50 | 2450 | 19 |
| Stem | Till+Com+Ash2 | 50 | 0.3 | <1.54 | 63.5 | 12 | 3350 | <0.1 | 0.324 | 3.08 | 44.6 | 3860 | 1160 | **196** | 302 | 0.555 | 755 | <1.54 | 1700 | 24 |
| Stem | Till+Com+Bc1+Ash1 | 50 | **0.5** | <1.52 | 37.4 | **47** | 3120 | <0.1 | <0.304 | 2.59 | 46.8 | **5100** | 1850 | 38 | **3450** | <0.304 | 594 | <1.52 | 1950 | 20 |
| Stem | Till+Com+Bc1+Ash2 | 50 | 0.3 | <1.54 | 48.9 | 14 | 3930 | <0.1 | <0.308 | 2.81 | 60.2 | 4270 | 1210 | 142 | 565 | 0.524 | 780 | <1.54 | 2100 | 26 |
| Stem | Till+Com+Bc3+Ash1 | 50 | 0.4 | <1.54 | 39.3 | **44** | 4390 | <0.1 | 0.554 | 2.63 | 57.2 | 4930 | 1440 | 66 | 2060 | 0.354 | 577 | <1.54 | 2700 | 19 |
| Stem | Till+Com+Bc3+Ash2 | 50 | 0.3 | <1.52 | 59.2 | 12 | 3340 | <0.1 | <0.305 | 2.67 | 53.1 | 4350 | 1550 | 160 | 399 | 0.442 | **877** | <1.52 | 2100 | 26 |
| Roots | Till | 50 | 0.6 | <1.53 | **905** | 11 | 6600 | 0.199 | **2.56** | 12.9 | 1470 | 5300 | 2010 | 117 | 560 | 3.83 | 1230 | <1.53 | 3650 | 28 |
| Roots | Till+Com | **51** | **0.7** | <1.54 | 680 | 13 | 4720 | 0.200 | 1.23 | 12.0 | 1240 | 5610 | 1740 | 121 | 723 | 2.07 | 1270 | <1.54 | 3080 | 34 |
| Roots | Tail | 50 | 0.6 | **2.06** | 186 | 16 | 5650 | 0.188 | 1.30 | **19.4** | 1210 | **5950** | **3720** | 44 | 692 | **18.6** | 959 | <1.56 | 3910 | 24 |
| Roots | Tail+Com | 49 | 0.6 | **3.48** | 206 | 20 | 5850 | 0.202 | 1.32 | **25.5** | **2750** | 5460 | **3030** | 50 | 732 | **20.6** | 1160 | <1.55 | 3560 | 20 |
| Roots | Till+Com+Bc1 | 49 | 0.6 | <1.54 | 496 | 12 | 8430 | **0.369** | 1.81 | 9.66 | 738 | 5480 | 1870 | 75 | 860 | 1.49 | 1330 | <1.54 | 6200 | 40 |
| Roots | Till+Com+Bc2 | 50 | **0.7** | <1.53 | 622 | 9.9 | 4600 | 0.138 | 1.81 | 12.0 | **2210** | 4660 | 1640 | 143 | 609 | 2.79 | 1220 | <1.53 | 3400 | 30 |
| Roots | Till+Com+Bc3 | 50 | 0.6 | <1.55 | **771** | 12 | 5770 | 0.218 | 1.65 | 14.1 | 1230 | 5400 | 1510 | 106 | 523 | 2.67 | 1390 | <1.55 | 3000 | 36 |
| Roots | Till+Com+Ash1 | 49 | 0.6 | <1.55 | 637 | **86** | 8720 | 0.248 | 1.83 | 11.1 | 1230 | 5620 | 2270 | 115 | 2020 | 2.42 | 1040 | <1.55 | 6610 | 32 |
| Roots | Till+Com+Ash2 | 50 | 0.6 | <1.54 | 637 | 11 | 4890 | 0.185 | **2.45** | 11.5 | 1300 | 4680 | 1570 | **156** | 532 | 3.95 | **1580** | <1.54 | 2780 | 32 |
| Roots | Till+Com+Bc1+Ash1 | 49 | 0.6 | <1.56 | 524 | **207** | **12900** | **0.437** | 1.73 | 11.6 | 1070 | 5200 | 2180 | 52 | **3160** | 1.62 | 932 | <1.56 | **7860** | **43** |
| Roots | Till+Com+Bc1+Ash2 | 50 | **0.7** | <1.55 | 524 | 14 | 6240 | 0.232 | 1.70 | 14.3 | 2340 | 5180 | 1610 | 113 | 869 | 2.80 | **1670** | <1.55 | 4060 | **40** |
| Roots | Till+Com+Bc3+Ash1 | 49 | 0.6 | <1.54 | 510 | **147** | **10600** | 0.324 | 1.70 | 11.4 | 1540 | 5910 | 1740 | 83 | 2410 | 1.56 | 966 | <1.54 | **6850** | 31 |
| Roots | Till+Com+Bc3+Ash2 | 50 | **0.7** | <1.56 | 627 | 11 | 4490 | 0.140 | 1.87 | 12.8 | 1610 | 5070 | 2050 | 113 | 817 | 3.27 | 1520 | <1.56 | 3230 | 38 |

**Supplementary Table 7.** Total element concentrations (mg kg^-1^) of white clover at harvest (from one combined sample. extractant: HNO_3_+H_2_O_2_, empty cells denote too small sample). Highest values are in bold to highlight the differences among the media.

| **Compartment** | **Medium** | **C** | **N** | **As** | **Al** | **B** | **Ca** | **Cd** | **Cr** | **Cu** | **Fe** | **K** | **Mg** | **Mn** | **Na** | **Ni** | **P** | **Pb** | **S** | **Zn** |
| --- | --- | --- | --- | --- | --- | --- | --- | --- | --- | --- | --- | --- | --- | --- | --- | --- | --- | --- | --- | --- |
| Roots | Till | 39 | 0.5 | <1.51 | **2850** | 2.9 | 5300 | <0.1 | **6.84** | 24.4 | 3890 | 9440 | 2290 | 188 | 815 | 7.02 | 1110 | 1.87 | 5040 | 27 |
| Roots | Till+Com | 40 | 0.5 | <1.53 | **2550** | 11 | 3690 | <0.1 | 5.70 | **26.0** | 3490 | 10600 | 2300 | 239 | 1020 | 5.19 | 1390 | <1.53 | 4030 | 31 |
| Roots | Tail | - | - | - | - | - | - | - | - | - | - | - | - | - | - | - | - | - | - | - |
| Roots | Tail+Com | **42** | 0.6 | **30.1** | 1550 | 8.4 | **11300** | 0.111 | 11.1 | **39.0** | **9110** | 13100 | 2660 | 184 | 408 | **68.7** | 1430 | <1.58 | 8360 | 23 |
| Roots | Till+Com+Bc1 | 41 | 0.6 | <1.58 | 2270 | 8.4 | 3800 | **0.205** | 4.49 | 24.9 | 2960 | 11000 | 1400 | 123 | 631 | 4.17 | 1590 | <1.58 | 2760 | 36 |
| Roots | Till+Com+Bc2 | 34 | 0.4 | <1.53 | 2360 | 5.9 | 3830 | <0.1 | **6.02** | 21.2 | 5440 | 10800 | 2110 | **275** | 697 | 5.18 | 1840 | 1.64 | 3890 | 34 |
| Roots | Till+Com+Bc3 | **42** | 0.6 | <1.56 | 1770 | 9.8 | 3520 | 0.109 | 3.83 | 22.9 | 2410 | 11000 | 1900 | 162 | 1110 | 3.85 | 1460 | 1.58 | 4120 | 33 |
| Roots | Till+Com+Ash1 | 38 | 0.5 | <1.50 | 1600 | **112** | 4050 | <0.1 | 4.01 | 14.0 | 2260 | 15000 | 2110 | 140 | 1030 | 3.53 | 1660 | <1.5 | 4710 | 22 |
| Roots | Till+Com+Ash2 | 37 | 0.5 | <1.53 | 1970 | 8 | 3190 | <0.1 | 4.40 | 20.1 | 3360 | 10700 | 1880 | 186 | 823 | 5.37 | 1740 | 1.90 | 3600 | 34 |
| Roots | Till+Com+Bc1+Ash1 | 40 | 0.6 | <1.50 | 1620 | **169** | 5810 | **0.210** | 4.30 | 16.6 | 2210 | **16100** | **3260** | 171 | **1830** | 3.06 | 1810 | 1.95 | 6850 | 34 |
| Roots | Till+Com+Bc1+Ash2 | 39 | 0.6 | <1.52 | 1790 | 11 | 4150 | 0.137 | 4.15 | 24.9 | 3420 | 11100 | 1700 | 155 | 760 | 4.09 | 1880 | 1.57 | 3840 | 40 |
| Roots | Till+Com+Bc3+Ash1 | 38 | **0.7** | <1.54 | 1390 | **172** | 7300 | 0.108 | 3.66 | 20.6 | 2330 | **16500** | **3920** | 159 | **1880** | 3.10 | 1820 | <1.54 | **9590** | 33 |
| Roots | Till+Com+Bc3+Ash2 | 41 | **0.7** | <1.54 | 1800 | 8.2 | 4000 | <0.1 | 4.70 | 29.6 | 3560 | 11000 | 1470 | 214 | 853 | 4.89 | **2030** | **2.03** | 3500 | **52** |
| Shoot | Till | 43 | 0.4 | <1.56 | **32.5** | 9.5 | 4070 | <0.1 | **0.937** | 4.17 | **105** | 12700 | **6230** | **485** | 894 | 2.00 | 999 | <1.56 | 8530 | 18 |
| Shoot | Till+Com | 43 | 0.5 | <1.57 | 14.4 | 51 | 4200 | <0.1 | 0.708 | 4.66 | 53.8 | 13900 | 5830 | **441** | 1110 | 1.29 | 1290 | <1.57 | 7730 | 18 |
| Shoot | Tail | - | - | - | - | - | - | - | - | - | - | - | - | - | - | - | - | - | - | - |
| Shoot | Tail+Com | **44** | 0.5 | <1.57 | 15.5 | 21 | **5070** | <0.1 | 0.361 | **6.79** | 71.4 | 17000 | **6200** | 337 | 250 | **3.90** | 1380 | <1.57 | **9120** | 19 |
| Shoot | Till+Com+Bc1 | **44** | **0.6** | <1.59 | 17.1 | 47 | 4350 | <0.1 | 0.413 | 4.94 | 56.6 | 16100 | 5040 | 344 | 853 | 0.969 | 1400 | <1.59 | 6060 | 23 |
| Shoot | Till+Com+Bc2 | **44** | 0.4 | <1.52 | 14.8 | 28 | 3180 | <0.1 | 0.335 | 3.13 | 52.8 | 14500 | 1910 | 223 | 137 | 0.669 | 1440 | <1.52 | 3160 | 8.8 |
| Shoot | Till+Com+Bc3 | 43 | 0.5 | <1.54 | 20.0 | 40 | 4240 | <0.1 | 0.479 | 4.63 | 53.8 | 15200 | 5050 | 355 | 988 | 0.957 | 1390 | <1.54 | 7330 | 19 |
| Shoot | Till+Com+Ash1 | 43 | 0.5 | <1.55 | 11.6 | **590** | 3210 | <0.1 | 0.357 | 3.40 | 41.8 | 19900 | 4510 | 212 | **2560** | 0.729 | **1970** | <1.55 | 5310 | 9.7 |
| Shoot | Till+Com+Ash2 | 43 | 0.5 | <1.58 | 11.8 | 36 | 3960 | <0.1 | 0.410 | 4.29 | 49.1 | 14200 | 4960 | **399** | 683 | 1.17 | 1410 | <1.58 | 6800 | 21 |
| Shoot | Till+Com+Bc1+Ash1 | 43 | 0.5 | <1.57 | 17.1 | **698** | 3230 | <0.1 | 0.409 | 3.13 | 42.6 | **21400** | 5070 | 160 | **2640** | 0.629 | 1870 | <1.57 | 5860 | 11 |
| Shoot | Till+Com+Bc1+Ash2 | **44** | 0.5 | <1.55 | 11.9 | 48 | 4190 | <0.1 | 0.792 | 4.38 | 38.7 | 15700 | 3830 | 281 | 676 | 0.823 | 1500 | <1.55 | 5240 | 20 |
| Shoot | Till+Com+Bc3+Ash1 | 43 | 0.5 | <1.56 | 17.5 | **637** | 3310 | <0.1 | 0.438 | 2.97 | 52.5 | **22300** | 4640 | 199 | **2520** | 0.547 | 1870 | <1.56 | 6410 | 10 |
| Shoot | Till+Com+Bc3+Ash2 | 43 | 0.5 | <1.55 | 12.8 | 50 | 4440 | <0.1 | 0.541 | 4.67 | 42.9 | 15700 | 4230 | 369 | 784 | 1.02 | 1630 | <1.55 | 6060 | **25** |

**Supplementary Table 8.** Total element concentrations (mg kg^-1^) of timothy at harvest (from one combined sample, extractant: HNO_3_+H_2_O_2_, empty cells denote too small sample). Highest values are in bold to highlight the differences among the media.

| **Compartment** | **Medium** | **C** | **N** | **As** | **Al** | **B** | **Ca** | **Cd** | **Cr** | **Cu** | **Fe** | **K** | **Mg** | **Mn** | **Na** | **Ni** | **P** | **Pb** | **S** | **Zn** |
| --- | --- | --- | --- | --- | --- | --- | --- | --- | --- | --- | --- | --- | --- | --- | --- | --- | --- | --- | --- | --- |
| Roots | Till | - | - | - | - | - | - | - | - | - | - | - | - | - | - | - | - | - | - | - |
| Roots | Till+Com | 43 | 1.9 | <1.53 | 717 | 23 | **7690** | 0.183 | 2.55 | 23.6 | 1400 | 21700 | **7170** | **499** | 1800 | 5.4 | 2570 | <1.53 | 9230 | 63 |
| Roots | Tail | - | - | - | - | - | - | - | - | - | - | - | - | - | - | - | - | - | - | - |
| Roots | Tail+Com | - | - | - | - | - | - | - | - | - | - | - | - | - | - | - | - | - | - | - |
| Roots | Till+Com+Bc1 | **44** | 2.3 | <1.55 | 617 | 19 | 4860 | 0.326 | 2.05 | 16.8 | 1050 | 16700 | 5800 | 228 | 2080 | 3.42 | 1710 | <1.55 | 6080 | 60 |
| Roots | Till+Com+Bc2 | 43 | 2.6 | <1.55 | 739 | 18 | 5550 | 0.14 | 2.49 | 18.7 | **1850** | 13900 | 4860 | 323 | 2740 | 5.44 | 2240 | <1.55 | 7310 | 52 |
| Roots | Till+Com+Bc3 | - | - | - | - | - | - | - | - | - | - | - | - | - | - | - | - | - | - | - |
| Roots | Till+Com+Ash1 | 42 | 3.0 | <2.24 | **830** | **103** | 5660 | 0.358 | 3.27 | 21.3 | 1320 | 27700 | 4440 | 272 | 2430 | 4.19 | 2610 | <2.24 | 8290 | 53 |
| Roots | Till+Com+Ash2 | 43 | 3.3 | <1.54 | 677 | 19 | 4610 | 0.139 | 2.34 | 21.3 | 1430 | 18500 | 5550 | 320 | **3160** | **6.83** | 4280 | <1.54 | 8400 | 85 |
| Roots | Till+Com+Bc1+Ash1 | 41 | 2.9 | 1.62 | **892** | 69 | 5370 | **0.574** | 3.14 | 18.9 | 1560 | 27200 | 4350 | 156 | 2370 | 4.59 | 2690 | <1.51 | 8360 | 69 |
| Roots | Till+Com+Bc1+Ash2 | **44** | **3.3** | <1.53 | 644 | 20 | 5230 | 0.336 | 2.78 | 22.7 | 1400 | 22300 | 4740 | 288 | 2750 | 5.70 | **4660** | <1.53 | 8720 | **90** |
| Roots | Till+Com+Bc3+Ash1 | 43 | 3.1 | **2.84** | 782 | 83 | 6070 | 0.403 | **3.68** | **26.0** | 1400 | **29600** | 5250 | 175 | 2750 | 4.06 | 2940 | <1.55 | **10300** | 71 |
| Roots | Till+Com+Bc3+Ash2 | 43 | 3.2 | <1.56 | 646 | 18 | 5260 | <0.1 | 3.02 | 18.9 | 1570 | 18800 | 4910 | 225 | **3410** | 5.79 | 4220 | <1.56 | 8450 | 76 |
| Shoot | Till | 38 | 0.6 | <1.91 | 89.4 | 42 | **28800** | <0.1 | 0.937 | **11.8** | 193 | 12600 | **13500** | **335** | 1780 | 4.53 | 1890 | <1.91 | **17300** | 27 |
| Shoot | Till+Com | 42 | 1.5 | <1.54 | 82.8 | 30 | 18100 | <0.1 | 0.617 | 6.34 | 213 | 20900 | 5550 | 292 | 952 | 1.65 | 1420 | <1.54 | 5770 | 19 |
| Shoot | Tail | - | - | - | - | - | - | - | - | - | - | - | - | - | - | - | - | - | - | - |
| Shoot | Tail+Com | 42 | 1.3 | <1.55 | 83.7 | 28 | 15300 | <0.1 | **1.19** | 7.80 | **445** | 20600 | 6320 | 47 | 509 | **5.37** | 1400 | <1.55 | 7460 | 12 |
| Shoot | Till+Com+Bc1 | 43 | 1.8 | <1.56 | 76.3 | 24 | 19300 | <0.1 | 0.405 | 6.00 | 177 | 18200 | 6310 | 170 | 929 | 1.28 | 1070 | <1.56 | 4310 | 25 |
| Shoot | Till+Com+Bc2 | 43 | 1.8 | <1.54 | 124 | 23 | 16500 | <0.1 | 0.924 | 6.36 | **430** | 15800 | 6670 | **380** | 1430 | 2.90 | 1510 | <1.54 | 4800 | 26 |
| Shoot | Till+Com+Bc3 | 42 | 1.7 | <1.51 | 86.7 | 28 | 17500 | <0.1 | 0.967 | 7.43 | 250 | 22100 | 5380 | 140 | 1030 | 1.66 | 1750 | <1.51 | 7360 | 24 |
| Shoot | Till+Com+Ash1 | 42 | **2.0** | <1.54 | **103** | **310** | 14600 | <0.1 | **1.08** | 6.90 | 237 | **30300** | 4680 | 124 | 2230 | 1.45 | 1640 | <1.54 | 5890 | 22 |
| Shoot | Till+Com+Ash2 | 43 | 1.9 | <1.53 | 64.3 | 24 | 13200 | <0.1 | 0.735 | 6.04 | 284 | 17000 | 6610 | 194 | 2380 | 2.22 | 2490 | <1.53 | 5400 | **29** |
| Shoot | Till+Com+Bc1+Ash1 | 42 | 1.7 | <1.52 | 52.5 | **223** | 14200 | <0.1 | 0.396 | 6.87 | 153 | 25500 | 5220 | 68 | 2120 | 1.11 | 1940 | <1.52 | 5020 | 23 |
| Shoot | Till+Com+Bc1+Ash2 | 42 | 1.8 | <1.54 | 55.8 | 26 | 15100 | <0.1 | 0.879 | 6.74 | 230 | 19300 | 6110 | 159 | 1400 | 1.47 | **2560** | <1.54 | 5180 | 28 |
| Shoot | Till+Com+Bc3+Ash1 | 42 | 1.9 | <1.53 | 59.3 | **248** | 13100 | <0.1 | 0.367 | 5.98 | 146 | 24900 | 5990 | 58 | **2450** | 1.06 | 1700 | <1.53 | 6070 | 22 |
| Shoot | Till+Com+Bc3+Ash2 | 43 | 1.9 | <1.55 | 73.6 | 23 | 11400 | <0.1 | 0.621 | 6.03 | 219 | 18500 | 5210 | 171 | 1800 | 1.66 | **2510** | <1.55 | 5280 | 24 |
